# A meta-analysis of Th1 and Th2 cytokine profiles differentiating tuberculous from malignant pleural effusion

**DOI:** 10.1038/s41598-022-06685-8

**Published:** 2022-02-17

**Authors:** Yulin Zeng, Liwei Wang, Hai Zhou, Yu Qi

**Affiliations:** 1 Department of Anesthesiology, Graduate School of Medical and Dental Sciences, Tokyo Medical and Dental University, Tokyo, Japan; 2 Department of Anesthesiology, Xuzhou Central Hospital, 199 Jiefang South Road, Xuzhou, 221009 China

**Keywords:** Lung cancer, Tuberculosis

## Abstract

To clarify the predominance of Th1 or Th2 immune responses in malignant and tuberculous pleural effusion (MPE and TPE, respectively), we performed a meta-analysis of previously published results of the levels of Th1/Th2 cytokines associated with these two types of pleural effusion to evaluate the use of Th1/Th2 cytokine profiles in distinguishing TPE from MPE. We searched the PubMed and EMBASE databases for studies indexed from 2000 to March 2021. We included studies that (a) diagnosed TPE and MPE based on culture or pleural tissue biopsy and that (b) compared levels of Th1/Th2 cytokines between TPE and MPE. Pooled data based on a random-effects model or fixed-effects model and standardized mean differences (SMDs) across studies were used to compare TPE and MPE. We also performed Egger’s test to assess publication bias. Of 917 identified studies, a total of 42 studies were selected for the meta-analysis. Compared with MPE subjects, TPE subjects had a significantly higher level of TNF-α [2.22, (1.60–2.84)], an elevated level of IFN-γ [3.30, (2.57–4.40)] in pleural effusion, a situation where the Th1 immune response dominated. Conversely, the levels of interleukin-4 (IL-4) and IL-10 (Th2 cytokines) were higher in the MPE subjects than in the TPE subjects, showing statistically nonsignificant tiny effects [−0.15, (−0.94 to 0.63) and −0.04, (−0.21 to 0.12), respectively]. We confirmed that TPE, a situation in which the Th1 cytokines are predominant. The slight preponderance of Th2 cytokines in MPE, which is not convincing enough to prove.

## Introduction

Malignant and tuberculous pleural effusion (MPE and TPE, respectively) are the two most common types of exudative pleural effusions, and both are associated with a typical accumulation of lymphocytes^1,2^. Naïve CD4 + T cells are activated by the antigen-MHC complex then differentiate into functional T-helper 1(Th1) or T-helper 2 (Th2) subsets^3^. Th1 cytokines include interleukin (IL)-2, tumor necrosis factor (TNF)-α, and interferon (IFN)-γ, whereas IL-4, IL-5, and IL-10 are considered Th2 cytokines^4^. IL-6 is an inflammatory cytokine produced by monocytes, macrophages, and dendritic cells^5,6^. IL-6 has emerged as an important regulator of Th1/Th2 differentiation, promoting the IL-4-dependent induction of Th2 differentiation and inhibiting Th1 differentiation by upregulating suppressor of cytokine signaling (SOCS)-1 expression^7^.

Immune responses mediated by either the Th1 or Th2 subset dominate depending on different types of pleural effusion. The Th1-dominated immune response is considered an important factor in the containment of mycobacteria, and the main immune effector mechanism involves classically activated macrophages that arise in response to Th1 cytokine signals^8^. Th1 cytokines have been shown to predominate in immunity to lung tuberculosis (TB)^9,10^. Notably, TPE is considered a common localized form of extrapulmonary TB. However, whether the mechanism of localized immune response in TPE is dominated by the Th1 or Th2 subset remains to be investigated. MPE, on the other hand, has been associated with Th2 cytokine predominance^11,12^. Although some reports have shown a bias towards Th2 predominance in MPE, some reports have not^13^.

Therefore, in this study, we performed a meta-analysis of all available studies to quantitatively evaluate the Th1/Th2 cytokine profiles in TPE and MPE as well as to assess the potentially diagnostic value of these cytokines in discriminating TPE from MPE.

## Materials and methods

This meta-analysis was carried out in accordance with the Preferred Reporting Items for Systematic Reviews and Meta-analysis (PRISMA) Statement^14^, and it had been registered with International Platform of Registered Systematic Review and Meta-analysis Protocols (No. INPLASY202210005). An approval from the institutional review board was not necessary, as we extracted only summary information from previously published articles.

### Search strategy

A systematic search was conducted (Zeng Y.L. and Qi Y.) of the PubMed and EMBASE databases from 2000 to March 2021. We selected eligible studies documenting the levels of pleural effusion about Th1/Th2 cytokine profiles. The following key words were used in the database search: malignant pleural effusion, tuberculous pleural effusion, tumor necrosis factor-alpha, TNF-α, interferon gamma, IFN-γ, interleukin 2, IL-2, interleukin 4, IL-4, interleukin 5, IL-5, interleukin 6, IL-6, interleukin 10, and IL-10. The details of the strategy are available in Supplementary Methods.

### Study selection and data extraction

The inclusion criteria were as follows: (1) original and human studies; (2) studies with a title or abstract including the terms “tuberculous and malignant pleural effusion” and “cytokines”; (3) studies reporting the pleural effusion levels of TNF-α, IFN-γ, IL-2, IL-4, IL-5, IL-6, and IL-10 in patients; (4) MPE diagnosed based on malignant cells in pleural fluid or pleural biopsy; (5) TPE diagnosed based on Ziehl–Neelsen staining or positive mycobacteria culture, or positive pleural biopsy tissue samples; and (6) studies available with full text. No limitation was applied regarding the histological type of MPE, stages of cancer, severities of the disease, or region and race of the study subjects. We extracted demographic details from each included study as follows: the country of origin, surname of the first author, year of publication, sample size, research design, and patient's age. The standard deviation (SD), median, and interquartile range (IQR) of the pleural effusion levels of Th1/Th2 cytokines were extracted for each potentially included study. We eliminated studies that were reviews, case reports, meta-analyses or conference abstracts; nonhuman experiments; non-English language studies; or studies that presented insufficient data for pooling. Data extraction was conducted by two independent investigators (Z.Y.L. and Z.H.) via a form created for this study. Divergences were resolved by consensus or by consulting a senior investigator.

### Quality evaluation

We used the Newcastle–Ottawa Scale (NOS) to assess the methodological quality of the studies describing differences in Th1/Th2 cytokine levels in TPE and MPE and considered any study with a score of 6 or more (the highest score was 9) as being of good quality. The results are displayed in Supplementary Methods. Two reviewers independently evaluated the quality of each study.

### Statistical analysis

The outcomes were all continuous variables: Th1/Th2 cytokine levels in TPE and MPE. Data provided as the mean and SD were extracted. Data provided as the median and IQR were transformed to the mean and SD according to the method of S.P. Hozo (2005)^15^. We used standardized mean differences (SMDs) and 95% confidence intervals (CIs) to assess the levels of Th1/Th2 cytokines in TPE compared to MPE. The Cochran’s Q test, *I*^*2*^ statistic and *P* value were used to assess the heterogeneity. A *P* ≥ 0.1 or *I*^*2*^ ≤ 50% was considered as no significant heterogeneity, and the fixed-effects model (FEM) was applied; otherwise, the random-effects model (REM) was used. We used the sensitivity analysis to assess the robustness. Subgroup analyses were used to stratify the studies by covariables including country, study design, etiology, publishing year and quality scores based on meta-regression, in order to explore potential sources of heterogeneity. The software RevMan 5.2 and STATA version 14.0 were used for the statistical analyses and images. Begg's test was used to assess the asymmetry of the funnel plot. *p* < 0.05 was considered publication bias, and if present, the trim-and-fill method was adopted to determine the influence of publication bias on the results.

## Results

### Characteristics of the included studies

**Figure 1:**
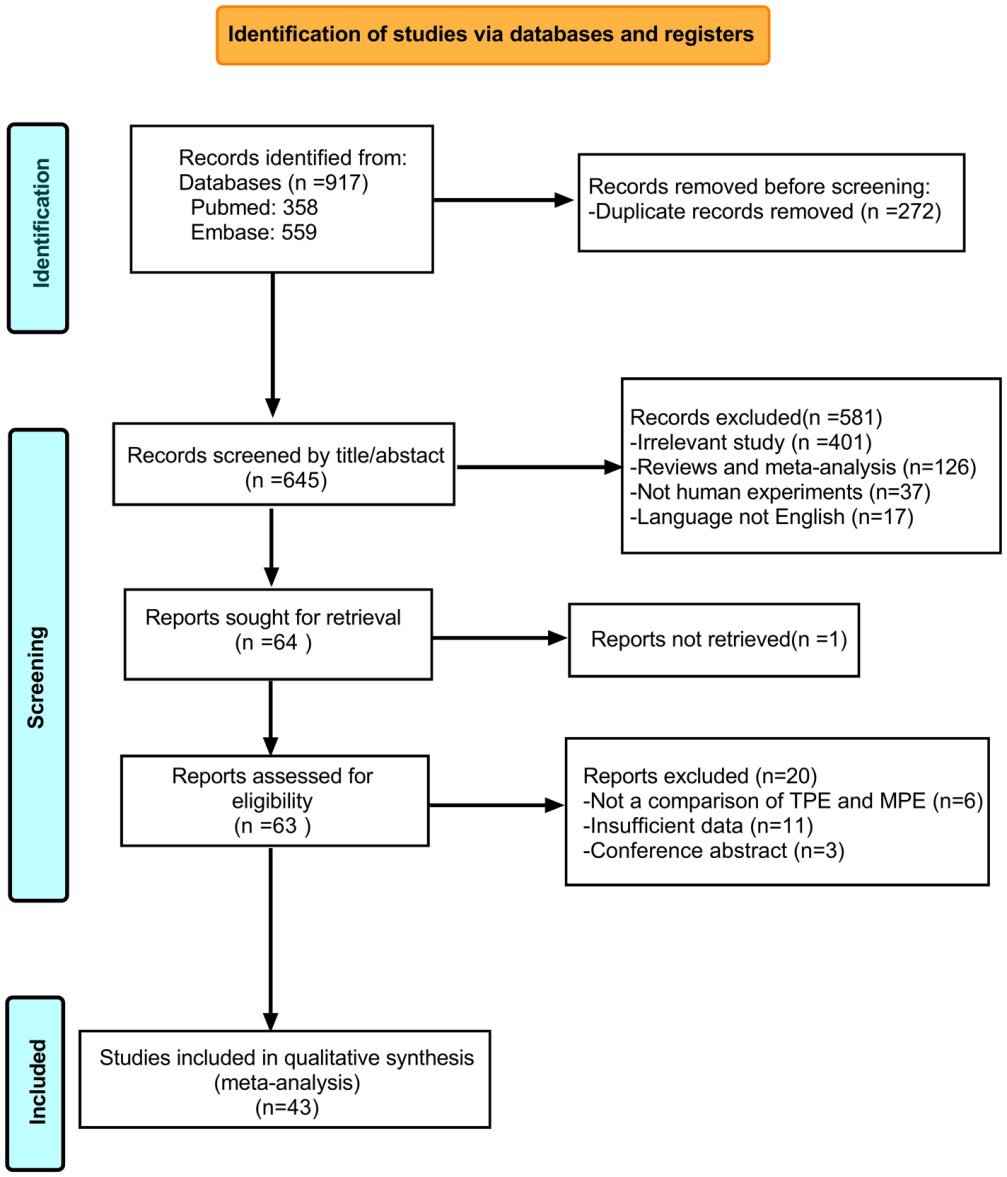
PRISMA flow chart of the selection of studies.

A total of 917 potentially relevant studies identified from the database were retrieved, of which 43 studies were included in the final meta-analytical processes. The study selection process is detailed in Fig. 1. In addition, we provide the details of the selected studies in Table 1. Considering the large number of included studies with information on TNF-α and IFN-γ, we only included the ELISA results, but other measurement methods [including cytometric bead array (CBA), chemiluminescent enzyme immunoassay (CLEIA), radioimmunoassay (RIA)] were included for assessments of the remaining cytokines. One study was excluded due to CBA measurements of IFN-γ levels^56^. The quality ratings for each study and characteristics of the included studies are presented in Supplementary Methods.

**Table 1:** Characteristics of the included original studies regarding the Th1/Th2 cytokine profiles for tuberculous and malignant pleural effusion.

Cytokines	No. of studies	No. of patients	Assay methodology	SMD (95%)	Model	Heterogeneity (%)		*p* value
						*I*^*2*^	*P*	
TNF-α	19^16–34^	TPE: 571 MPE: 661	ELISA (N = 19)	2.22	Random	95	< 0.001	< 0.001
IFN-γ	20^11,13,17,18,20,29,33,35–47^	TPE: 728 MPE: 992	ELISA (N = 20)	3.82	Random	97	< 0.001	< 0.001
IL-2	3^35,48,49^	TPE: 179 MPE: 210	ELISA (N = 1) Others (N = 2)	-0.07	Fixed	46	0.16	0.47
IL-4	6^13,20,35,40,49,50^	TPE:201 MPE:288	ELISA (N = 4) Others (N = 2)	-0.15	Random	92	< 0.001	0.70
IL-6	12^22,27,32,39,48–55^	TPE: 361 MPE: 731	ELISA (N = 7) Others (N = 5)	3.53	Random	98	< 0.001	< 0.001
IL-10	8^11,13,20,21,29,48–50^	TPE: 280 MPE: 549	ELISA (N = 5) Others (N = 3)	0.17	Random	66	0.005	0.25

### Th1/Th2 cytokine levels in TPE and MPE

#### Analysis of TNF-α

**Figure 2:**
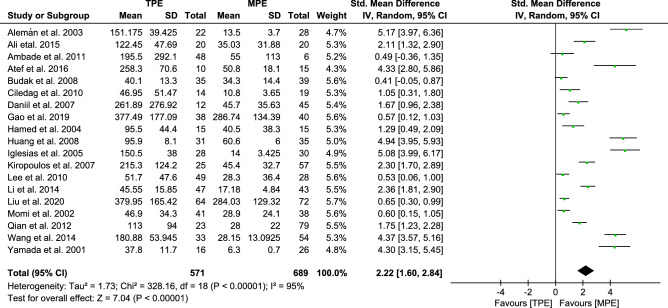
Forest plot of 19 studies comparing TNF-α levels in TPE and MPE subjects. The standardized mean difference (the pleural effusion levels of TNF-α in TPE subjects minus that in MPE subjects) was estimated by meta-analysis.

In a pooled analysis of all 19 trials, the results revealed that there was a significant increase in the pleural effusion levels of TNF-α in TPE subjects compared to MPE subjects [2.22, (1.60–2.84, *p* < 0.00001)] (Fig. 2). However, there was statistically significant heterogeneity among the studies (*I*^*2*^ 95%). Subsequently, sensitivity analysis (Fig. S1) was conducted by excluding studies one by one at a time, but neither the magnitude nor the direction of the effect size was substantially altered. We showed that the pooled SMD was stable and reliable, implying higher TNF-α levels in TPE. To explore the sources of the heterogeneity, we performed meta-regression analysis based on subgroup stratification by variables, including country, design, etiology, publishing year and quality scores. In the stratified analyses (Table S1), there was no reliable evidence that the above variables represented the main source of heterogeneity. Therefore, we considered that the heterogeneity might be due to differences in ELISA manufacturers and the severity of the diseases selected for study. In this analysis, there was publication bias based on Begg's test (*p* < 0.05). We therefore used the trim-and-fill method to adjust for publication bias and examined its effect on the pooled SMD (Supplementary Methods), yielding an adjusted effect (SMD = 1.038, 95% CI 0.328–1.748, *p* < 0.01).

#### Analysis of IFN-γ

**Figure 3:**
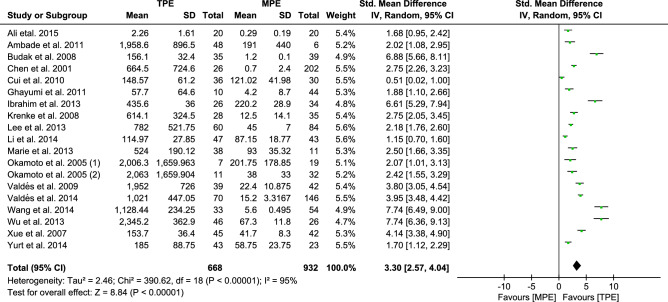
Forest plot of 19 studies comparing IFN-γ levels in TPE and MPE subjects. The standardized mean difference (the pleural fluid levels of IFN-γ in TPE subjects minus that in MPE subjects) was estimated by meta-analysis.

Pooled analysis of all 20 trials revealed that there was a significant increase in the pleural effusion levels of IFN-γ in TPE subjects compared to MPE subjects [3.82, (2.97–4.66, *p* < 0.00001)] (Fig. S2), with statistically significant between-study heterogeneity (*I*^*2*^ 97%). Subsequently, we performed sensitivity analysis, which indicated that there was considerable variation in one trial^47^. After removing that trial, the remaining results were stable through sensitivity analysis, the REM of pooled SMDs [3.30, (2.57–4.40, *p* < 0.00001)] (Fig. 3). Our pooled data suggested higher IFN-γ levels in TPE. However, despite a decrease in heterogeneity (*I*^*2*^ from 97 to 95%) with the removal of that trial, substantial heterogeneity still existed. To explore possible sources of heterogeneity, we further performed meta-regression analysis based on subgroup stratification by country, design, etiology, publishing year and quality scores. In the stratified analyses (Table S2), there was no reliable evidence that the above variables were the main source of heterogeneity; however, Egger's test indicated evidence of publication bias (*p* < 0.05). The trim and fill method was used to impute 3 missing studies to the left of the funnel plot to ensure symmetry, and the REM of pooled SMDs was adjusted [2.567, 95% CI 1.750–3.384, *p* < 0.001] (Supplementary Methods).

#### Analysis of IL-2

**Figure 4:**
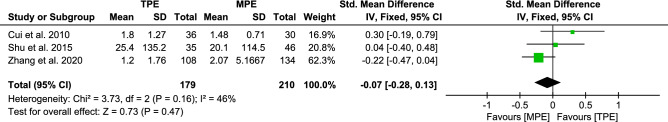
Forest plot of 3 studies comparing IL-2 levels in TPE and MPE subjects. The standardized mean difference (the pleural effusion levels of IL-2 in TPE subjects minus that in MPE subjects) was estimated by meta-analysis.

As can be seen in Fig. 4, although pooled analysis of all 3 included trials revealed that the FEM of pooled SMDs [−0.07, (−0.28 to 0.13, *p* < 0.00001)] suggested that IL-2 levels were higher in MPE subjects than TPE subjects, these differences were not statistically significant [*p* = 0.47 > 0.05]. The reason for this result is probably due to the small number of included studies. An assessment of publication bias was not accomplished because fewer than ten trials were included.

#### Analysis of IL-4

**Figure 5:**
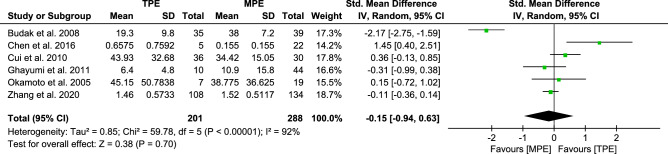
Forest plot of 6 studies comparing IL-4 levels in TPE and MPE subjects. The standardized mean difference (the pleural effusion levels of IL-4 in TPE subjects minus that in MPE subjects) was estimated by meta-analysis.

Pooling the data from the six trials revealed that the REM of pooled SMDs [−0.15, (−0.94 to 0.63)] had statistically significant between-trial heterogeneity (*I*^*2*^ 92%); the results are presented in Fig. 5. Although MPE subjects had higher levels of IL-4 than TPE subjects, this difference was not statistically significant (*p* = 0.70 > 0.05).

#### Analysis of IL-10

**Figure 6:**
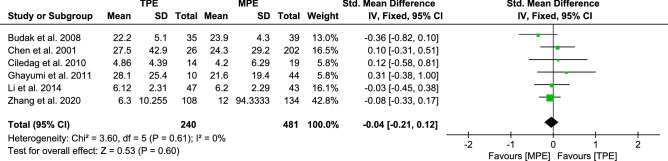
Forest plot of 6 studies comparing IL-10 levels in TPE and MPE subjects. The standardized mean difference (the pleural effusion levels of IL-10 in TPE subjects minus that in MPE subjects) was estimated by meta-analysis.

A meta-analysis of the data from eight trials on IL-10 was performed as shown in Figure S3a, the REM of pooled SMDs [0.17, (−0.12 to 0.46)]. However, there was statistically significant heterogeneity among the above results (*I*^*2*^ 66%, *p* < 0.00001). We excluded two studies^48,50^ according to the results of the Galbraith radial plot (Fig. S3b), and the heterogeneity was decreased appreciably (*I*^2^ from 66 to 0%) as shown in Fig. 6. On the basis of the 6 remaining trials, the FEM of pooled SMDs [−0.04, (−0.21 to 0.12)] suggested higher IL-10 levels in MPE subjects.

#### Analysis of IL-6 between TPE and MPE

**Figure 7:**
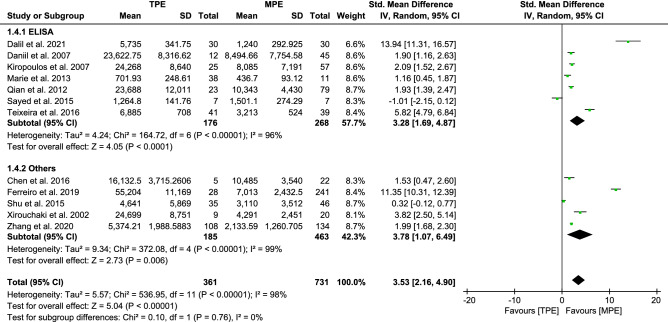
Forest plot of 12 studies comparing IL-6 levels in TPE and MPE subjects. The standardized mean difference (the pleural fluid levels of IL-6 in TPE subjects minus that in MPE subjects) was estimated by meta-analysis.

IL-6 was the most commonly measured inflammatory cytokine. On the basis of 12 trials, the REM of pooled SMDs [3.53, (2.16–4.90)] indicated substantial heterogeneity between studies (*I*^2^ = 98%, *p* < 0.00001) as shown in Fig. 7. The pooled results confirmed that the levels of IL-6 in TPE patients were higher than those in MPE patients. To assess the stability of the pooled results of the meta-analysis of IL-6, sensitivity analysis was conducted by excluding studies one by one at a time. Neither the magnitude nor the direction of the effect size was substantially altered, indicating that our results were robust. Furthermore, we excluded two trials^22,53^ based on the sensitivity analysis (Fig. S4a). After the deletion of these two trials, the remaining results were stable through sensitivity analysis (Fig. S4b) and caused no appreciable change in the pooled SMDs [1.92, (1.13–2.71)], but the heterogeneity was decreased (*I*^*2*^ from 98 to 94%) (Fig. S4c). To test the robustness of our results, we perform stratified analyses to detect the sources of the heterogeneity in this study as shown in Table S3. Among the 12 trials, 7 presented ELISA measurements, and the remaining five reported other measurements (2 with CBA results, 2 with RIA results, and 1 with CLEIA results). Consequently, we considered that the main heterogeneity arises from differences in measurement methods. However, nonsignificant differences in between-group heterogeneity were found in some stratified analyses, and measurement methods did not indicate additional sources of heterogeneity. In this analysis, there was no publication bias based on Egger’s test (*p* = 0.150, Supplementary Methods).

## Discussion

To the best of our knowledge, this is the first large-scale meta-analysis to systematically investigate the levels of Th1/Th2 cytokines in MPE and TPE. Our results showed that the Th1 cytokine profile was predominant in TPE compared to MPE. We confirmed that the immune response in TPE is dominated by Th1 polarization. Although significant heterogeneity was detected, trials with extreme SMDs were removed based on sensitivity analysis and Galbraith radial plot assessment, which diminished the heterogeneity and provided more stable results. Despite the slight bias toward Th2 cytokines in MPE subjects, the pooled SMD was not statistically significant.

Exudative pleural effusion results from a vicious loop of interactions between the immune system and a pleural or parenchymal disease like TB infection, malignancy or inflammation. It results in abnormal accumulation of fluid in the pleural space via altering the permeability of the pleural membranes to enhance plasma extravasation. TB and malignant disease are among the most frequent causes of exudative pleural effusions. Th cells are known to play important roles in the pathogenesis of exudative pleural effusion and host defense.

Th1 cells have been observed to have an anti-infectious role in TB^57^. Th1-mediated host immunity inhibits further multiplication of *Mycobacterium tuberculosis* (Mtb) via the secretion of IFN-γ and other Th1 cytokines, which activate macrophages, promote phagosome maturation to stimulate phagocytosis, and stimulate the production of reactive nitrogen intermediates^58^. TNF-α is essential for stimulating the chemotaxis of inflammatory cells to sites of infection and leads to granulomatous response to containment disease progression^59^. Normally, IL-6 is a proinflammatory cytokine, inducing the production of acute phase proteins to promote inflammation. Nevertheless, macrophages infected with Mtb induce the production of IL-6, which inhibits the response of macrophages to IFN-γ, resulting in the inability to eradicate Mtb infection^60^. The increased IL-6 levels also contribute to increased expression of suppressor of cytokine signaling (SOCS) in TB^61^. SOCS1 is regarded as an important mediator that inhibits IFN-γ secretion by macrophages, which in turn hampers the early clearance of Mtb by macrophages^62^. Consistent with the results of other studies, our study found higher levels of IL-6 in TPE subjects, which suggested that IL-6, as an important cytokine, may be involved in the formation of TPE and play an important role in the occurrence and development of pulmonary tuberculosis (PTB).

Different from Th1 cytokines, IL-10 is considered an inhibitory and anti-inflammatory Th2 cytokine that negatively regulates IFN-γ-mediated host immunity, limits pathogen clearance in the early immune response to Mtb and mediates long-term chronic infection^63^. The IFN-γ/IL-10 ratio is a useful objective marker of the clinical severity of TB^64^. Additionally, IL-4 is a classic Th2 cytokine that may subvert mycobacterial containment in macrophages by perturbating Th1-related pathways and regulatory T cells (Tregs)^65^.

Early studies have suggested that Th1- and Th2-cell-producing cytokines play important roles in the immune microenvironment of tumors; Th1 cytokines relevant to antitumor immunity have been reported, whereas Th2 cytokines are related to tumor invasiveness and metastasis^66^. Notably, in advanced cancer, a shift from Th1 to Th2 cells in the tumor environment is often observed, and impaired Th1 cell-mediated immunity has been reported to be associated with cancer progression^67,68^. Anthony et al. reported that patients with advanced squamous cell carcinoma had a diminished Th1 antitumor immune response but stronger underlying Th2 immune response^69^. This shift was further demonstrated in malignant effusions^70^. Accordingly, these studies collectively indicate that a shift from a Th1 to a Th2 cell response in the microenvironment of tumors may play a crucial role in the development and progression of tumors. Moreover, evidence indicates that tumor-derived TGF-β-induced overexpression of IL-10 may drive the shift in the Th1/Th2 balance toward a Th2 response and inhibit the Th1 response^71^. The balance of Th1/Th2 cytokines in MPE subjects remains controversial. We speculate that the shift from Th1 to Th2 along with tumor progression may explain the discrepancy regarding whether Th2 cytokines predominate in MPE subjects, but this needs to be studied further.

As future studies clarify the roles of Th1/Th2 cytokine profiles and their effect on the local immune response, new therapeutic approaches to immunotherapy may be developed. Currently, a useful cytokine/anti-cytokine therapy involves supplementation of anti-inflammatory recombinant cytokines as well as neutralizing cytokines or antagonizing receptors by monoclonal antibodies (mAbs). Recombinant IL-2 has been used successfully for cancer therapy and was approved by the FDA for the treatment of metastatic renal-cell carcinoma in 1992 and for metastatic melanoma in 1998. Infliximab, a chimeric anti-TNFα monoclonal antibody, has been demonstrated to be an effective therapy for rheumatoid arthritis, Crohn's disease, ankylosing spondylitis and other autoimmune diseases. Despite these beneficial effects of infliximab treatment in autoimmune diseases, it carries the risk of TB occurrence and latent TB reactivation^72^. Based on the immunosuppressive ability of IL-10 in tumor immunity, therapeutic strategies targeting the IL-10 signaling pathway have evolved considerably in cancer immunotherapy^73^. In addition, overexpressed IL-4 and IL-13 receptors on cancer cells are specific targets for receptor-directed cancer immunotherapy^74^. Our study confirms changes in the Th1/Th2 cytokine profiles of patients with TPE and MPE, which may be helpful for future investigations of cytokine therapies.

Moreover, combining multiple biomarkers has previously been shown to enhance diagnostic accuracy. The combination of TNF-α and adenosine deaminase 2 (ADA_2_) has been shown to improve the specificity and accuracy of diagnosis for the differentiation of TPE from MPE^29^. Future studies are needed to determine if Th1/Th2 cytokine profiles confer supplementary diagnostic value for pleural effusions.

There are some unavoidable limitations of our study that are deserving of attention. First, significant heterogeneity was observed in some of our results; although stratified analyses were done, we did not identify the source of heterogeneity. Second, some of the trials provided only the median and IQR levels of the cytokines, which we converted to the mean and SD by Hozo et al.’s method, which might lead to slightly skewed data. Third, the sample sizes included in studies on Th2 cytokines were relatively small, and only six trials were included in the pooled results. Fourth, we did not examine the effect of the stages of cancer on cytokine levels in the included studies, which might lead to heterogeneity and deviation in this analysis. Fifth, few studies have reported the primary tumor causing MPE and the histological type of the primary tumor; therefore, future studies should investigate its effect on cytokine levels.

## Conclusions

In summary, this systematic review and meta-analysis confirms that pleural effusions caused by TB, a situation in which the Th1 cytokines are predominant. Although MPE appears to be slightly bias toward Th2 cytokines, this finding was not sufficiently convincing to prove. Our findings suggest that these cytokines are likely to serve supplementary diagnostic value for the differentiation of TPE from MPE. Moreover, it is important to know the role of cytokines derived from Th cells on the pathogenesis of diseases and host defense, which may provide new clues for cytokine immunotherapies.

## Supplementary Information

Supplementary Figures.

Supplementary Information.

Supplementary Tables.

## Data Availability

The data sets supporting the results of this article are included within the article and its supplementary material.
