# Supplementary material for: A meta-analysis of Th1 and Th2 cytokine profiles differentiating tuberculous from malignant pleural effusion
Source: Sci Rep. 2022 Feb 17;12:2743. doi: 10.1038/s41598-022-06685-8 (PMC8854582; doi:10.1038/s41598-022-06685-8)
Supplement: Supplementary file 1 — Supplementary Figures. [file 41598_2022_6685_MOESM1_ESM.pdf]

Supplementary Fig. S1

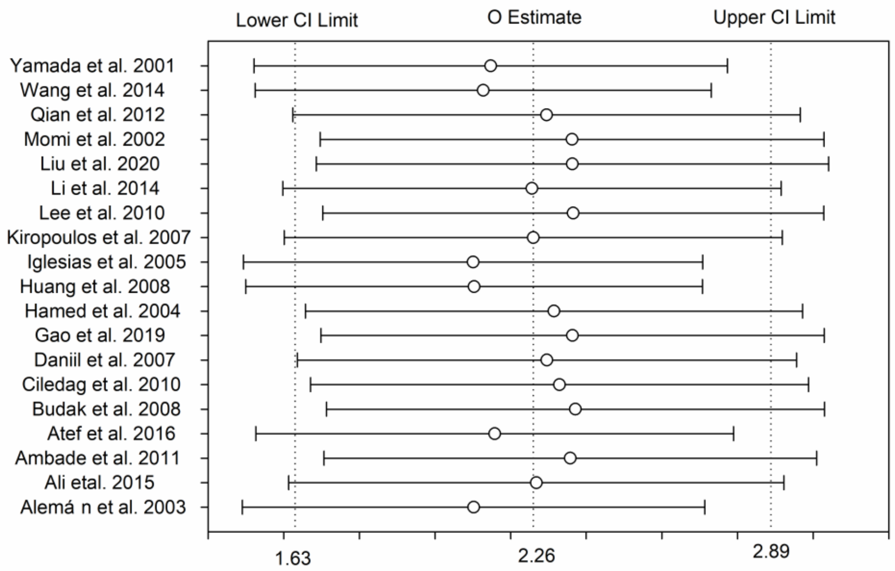

Sensitivity analysis of the all 19 studies on TNF-α included in the meta-analysis. The circle indicates the total combined effect SMD, the two ends of every broken line represent the respective 95% confidence interval.

Fig. S2a

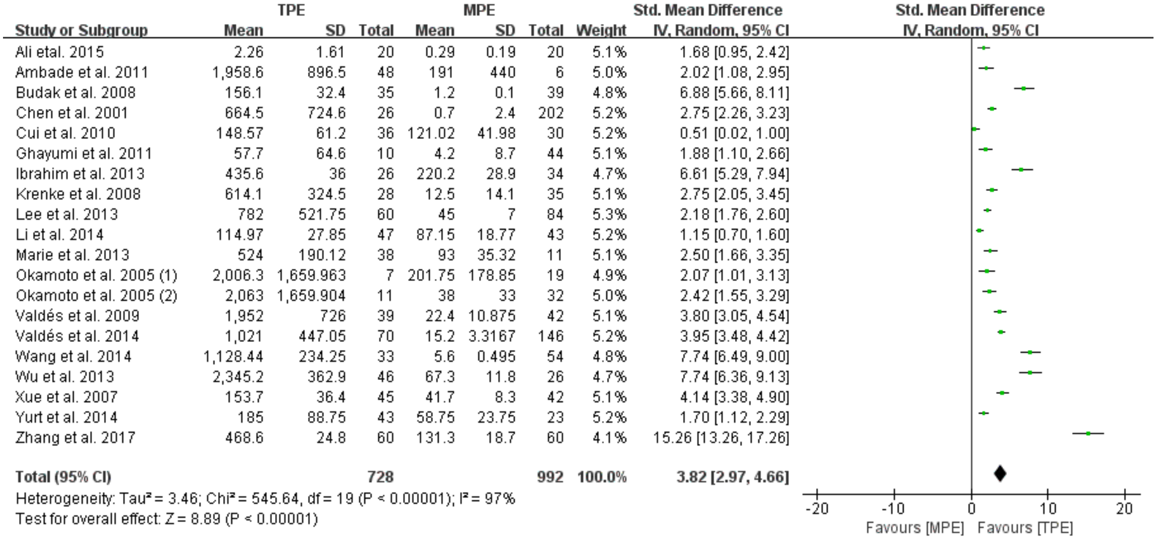

	TPE			MPE				Std. Mean Difference
Study or Subgroup	Mean	SD	Total	Mean	SD	Total	Weight	IV, Random, 95% CI
Ali etal. 2015	2.26	1.61	20	0.29	0.19	20	5.1%	1.68 [0.95, 2.42]
Ambade et al. 2011	1,958.6	896.5	48	191	440	6	5.0%	2.02 [1.08, 2.95]
Budak et al. 2008	156.1	32.4	35	1.2	0.1	39	4.8%	6.88 [5.66, 8.11]
Chen et al. 2001	664.5	724.6	26	0.7	2.4	202	5.2%	2.75 [2.26, 3.23]
Cui et al. 2010	148.57	61.2	36	121.02	41.98	30	5.2%	0.51 [0.02, 1.00]
Ghayumi et al. 2011	57.7	64.6	10	4.2	8.7	44	5.1%	1.88 [1.10, 2.66]
Ibrahim et al. 2013	435.6	36	26	220.2	28.9	34	4.7%	6.61 [5.29, 7.94]
Krenke et al. 2008	614.1	324.5	28	12.5	14.1	35	5.1%	2.75 [2.05, 3.45]
Lee et al. 2013	782	521.75	60	45	7	84	5.3%	2.18 [1.76, 2.60]
Li et al. 2014	114.97	27.85	47	87.15	18.77	43	5.2%	1.15 [0.70, 1.60]
Marie et al. 2013	524	190.12	38	93	35.32	11	5.1%	2.50 [1.66, 3.35]
Okamoto et al. 2005 (1)	2,006.3	1,659.963	7	201.75	178.85	19	4.9%	2.07 [1.01, 3.13]
Okamoto et al. 2005 (2)	2,063	1,659.904	11	38	33	32	5.0%	2.42 [1.55, 3.29]
Valdés et al. 2009	1,952	726	39	22.4	10.875	42	5.1%	3.80 [3.05, 4.54]
Valdés et al. 2014	1,021	447.05	70	15.2	3.3167	146	5.2%	3.95 [3.48, 4.42]
Wang et al. 2014	1,128.44	234.25	33	5.6	0.495	54	4.8%	7.74 [6.49, 9.00]
Wu et al. 2013	2,345.2	362.9	46	67.3	11.8	26	4.7%	7.74 [6.36, 9.13]
Xue et al. 2007	153.7	36.4	45	41.7	8.3	42	5.1%	4.14 [3.38, 4.90]
Yurt et al. 2014	185	88.75	43	58.75	23.75	23	5.2%	1.70 [1.12, 2.29]
Zhang et al. 2017	468.6	24.8	60	131.3	18.7	60	4.1%	15.26 [13.26, 17.26]
**Total (95% CI)**			**728**			**992**	**100.0%**	**3.82 [2.97, 4.66]**

Heterogeneity: Tau² = 3.46; Chi² = 545.64, df = 19 (P < 0.00001); I² = 97%
Test for overall effect: Z = 8.89 (P < 0.00001)

Forest plot for remaining 20 studies comparing IFN-γ levels in tuberculous pleural effusions and malignant effusions.

Supplementary Fig. S2b

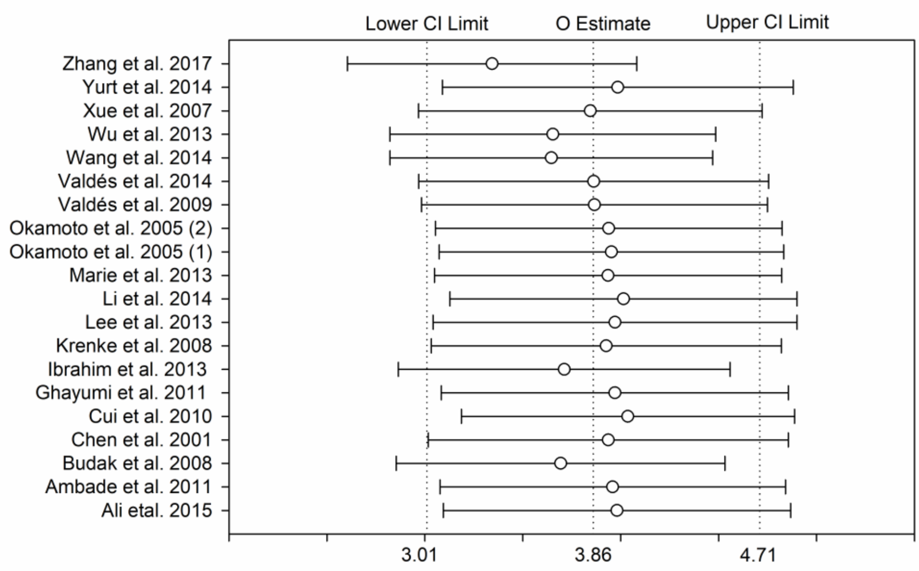

Sensitivity analysis of the all 20 studies on IFN-γ included in the meta-analysis.

Supplementary Fig. 2c

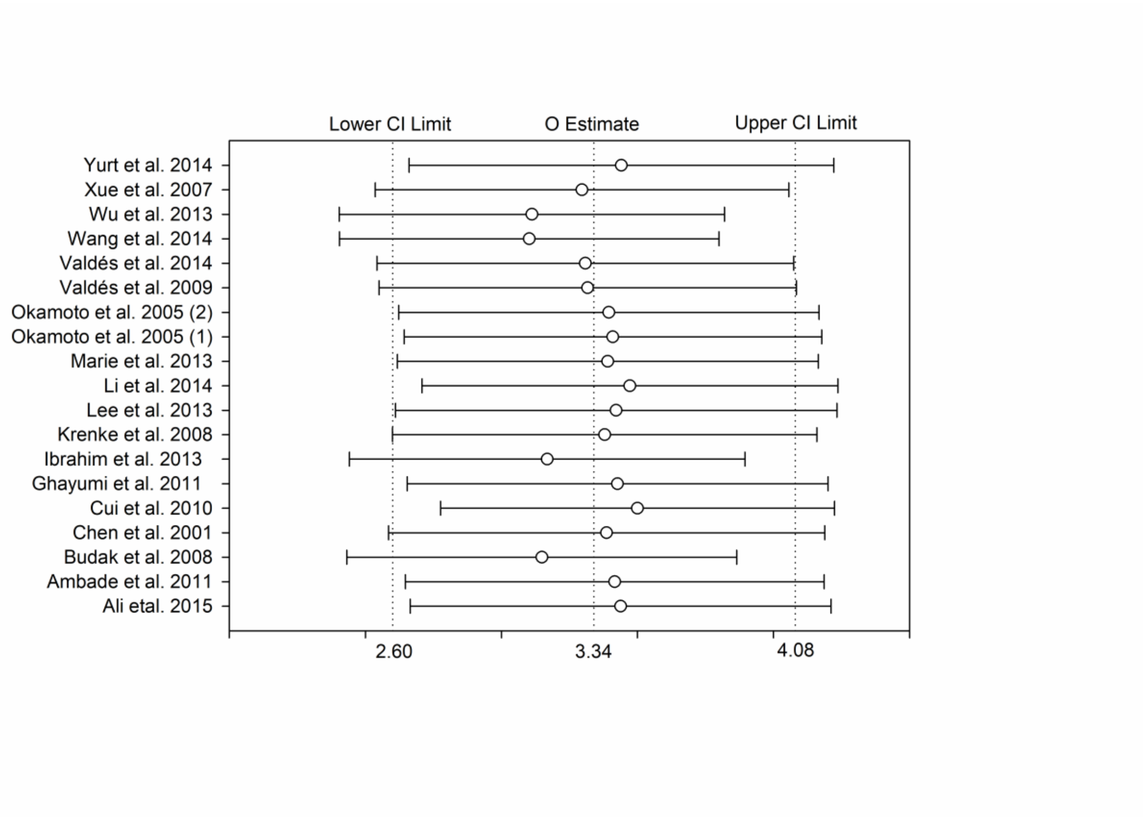

Sensitivity analysis of the remaining 19 studies on IFN-γ included in the meta-analysis.

Fig. S3a

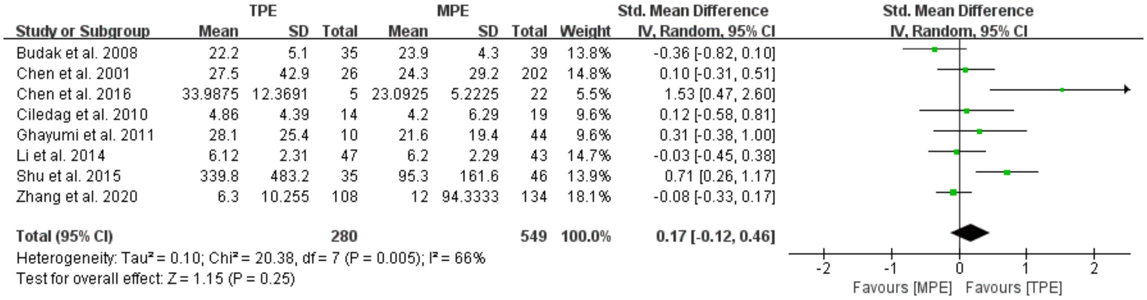

Forest plot for combined total 8 studies comparing IL-10 levels in tuberculous pleural effusions and malignant effusions.

Fig. S3b

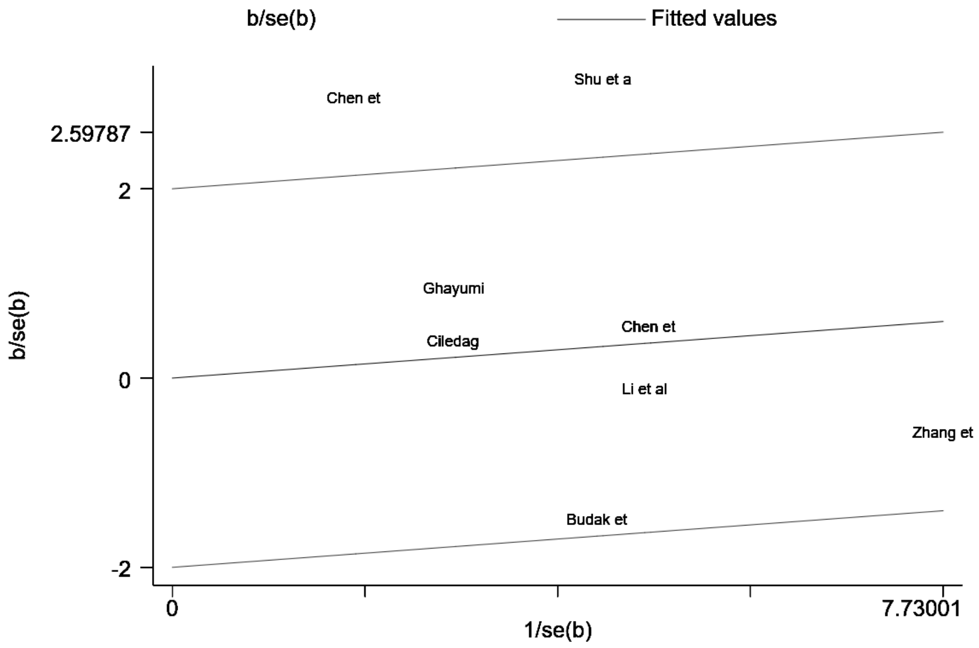

Galbraith radial plot for the heterogeneity across studies on IL-10.

Fig. S4a

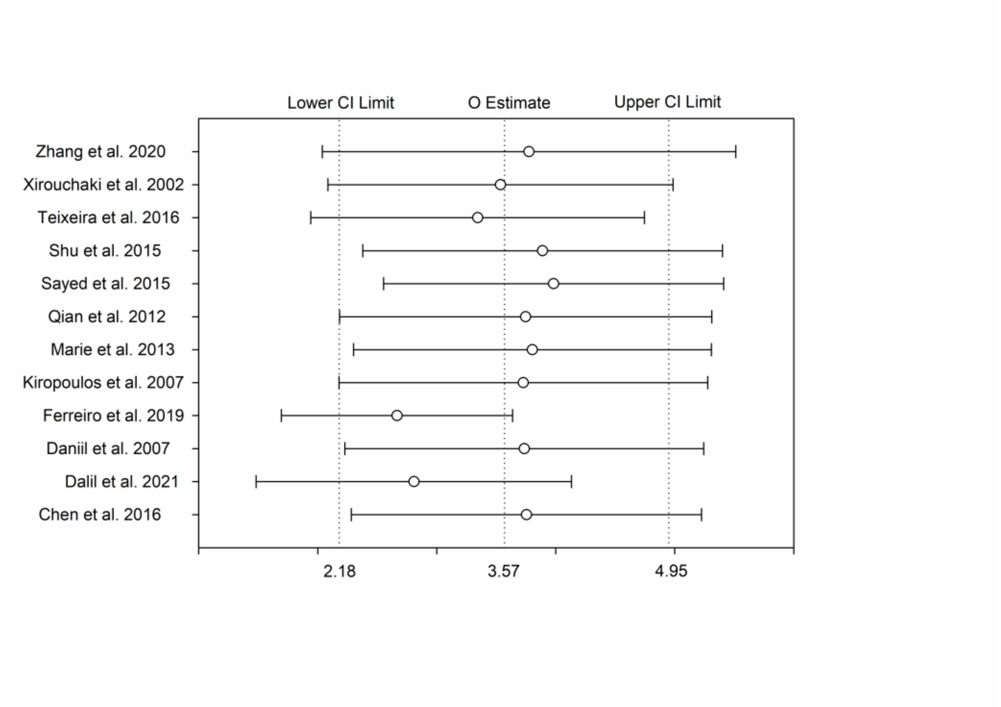

Sensitivity analysis of the all 12 studies on IL-6 included in the meta-analysis.

Fig. S4b

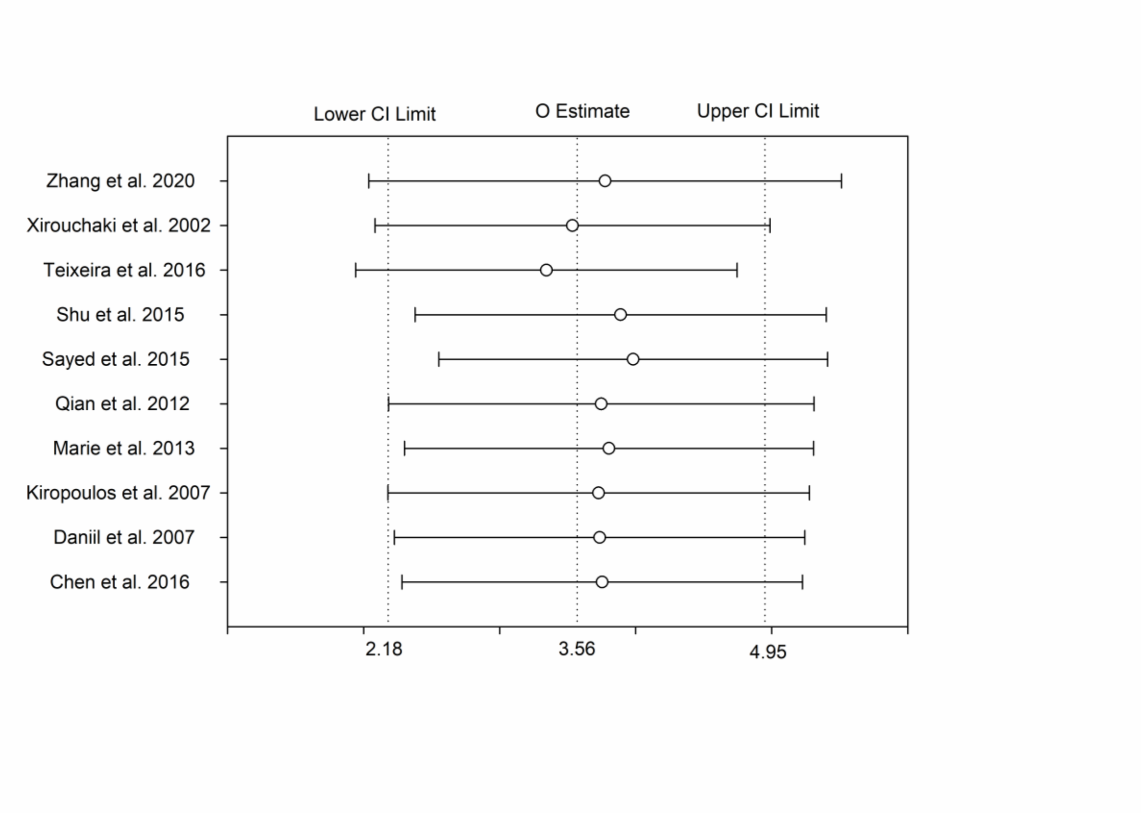

Sensitivity analysis of the remaining 10 studies on IL-6 included in the meta-analysis.

Fig. S4c

Study or Subgroup	TPE Mean	TPE SD	TPE Total	MPE Mean	MPE SD	MPE Total	Weight	Std. Mean Difference IV, Random, 95% CI
Chen et al. 2016	16,132.5	3,715.2606	5	10,485	3,540	22	9.3%	1.53 [0.47, 2.60]
Daniil et al. 2007	23,622.75	8,316.62	12	8,494.66	7,754.58	45	10.2%	1.90 [1.16, 2.63]
Kiropoulos et al. 2007	24,268	8,640	25	8,085	7,191	57	10.6%	2.09 [1.52, 2.67]
Marie et al. 2013	701.93	248.61	38	436.7	93.12	11	10.3%	1.16 [0.45, 1.87]
Qian et al. 2012	23,688	12,011	23	10,343	4,430	79	10.7%	1.93 [1.39, 2.47]
Sayed et al. 2015	1,264.8	141.76	7	1,501.1	274.29	7	9.1%	-1.01 [-2.15, 0.12]
Shu et al. 2015	4,641	5,869	35	3,110	3,512	46	10.8%	0.32 [-0.12, 0.77]
Teixeira et al. 2016	6,885	708	41	3,213	524	39	9.4%	5.82 [4.79, 6.84]
Xirouchaki et al. 2002	24,699	8,751	9	4,291	2,451	20	8.5%	3.82 [2.50, 5.14]
Zhang et al. 2020	5,374.21	1,988.5883	108	2,133.59	1,260.705	134	11.0%	1.99 [1.68, 2.30]
**Total (95% CI)**			**303**			**460**	**100.0%**	**1.92 [1.13, 2.71]**

Heterogeneity: Tau² = 1.46; Chi² = 139.51, df = 9 (P < 0.00001); I² = 94%
Test for overall effect: Z = 4.76 (P < 0.00001)

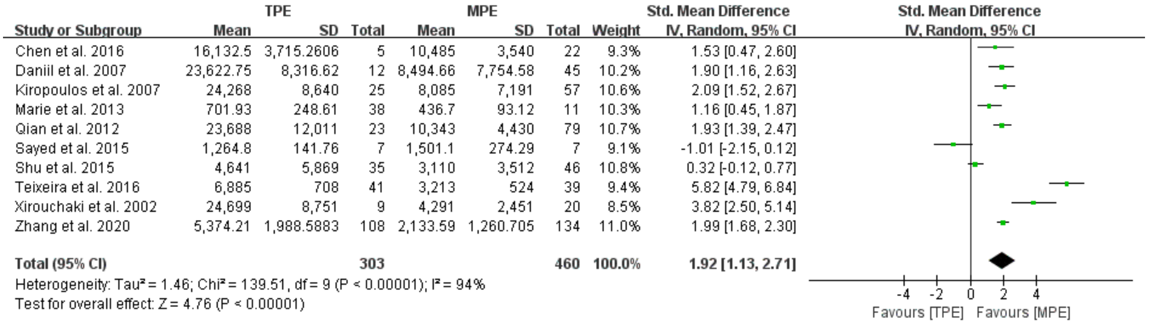

Forest plot for remaining 10 studies comparing IL-6 levels in tuberculous pleural effusions and malignant effusions.
