# Supplementary material for: A meta-analysis of Th1 and Th2 cytokine profiles differentiating tuberculous from malignant pleural effusion
Source: Sci Rep. 2022 Feb 17;12:2743. doi: 10.1038/s41598-022-06685-8 (PMC8854582; doi:10.1038/s41598-022-06685-8)
Supplement: Supplementary file 2 — Supplementary Information. [file 41598_2022_6685_MOESM2_ESM.pdf]

1. **Search strategy:**

Pubmed: ((("Pleural Effusion, Malignant"[Mesh]) OR ((((((Effusion, Malignant Pleural[Title/Abstract])) OR (Effusions, Malignant Pleural[Title/Abstract])) OR (Malignant Pleural Effusion[Title/Abstract])) OR (Malignant Pleural Effusions[Title/Abstract])) OR (Pleural Effusions, Malignant[Title/Abstract]))) OR ((("Pleural Effusion"[Mesh]) OR (((Effusion, Pleural[Title/Abstract]) OR (Effusions, Pleural[Title/Abstract])) OR (Pleural Effusions[Title/Abstract]))) AND (tuberculous))) AND ((((((("Interleukin-2"[Mesh]) OR (((((((((((((((Interleukin 2[Title/Abstract]) OR (IL-2[Title/Abstract])) OR (IL2[Title/Abstract])) OR (TCGF[Title/Abstract])) OR (Interleukine 2[Title/Abstract])) OR (Lymphocyte Mitogenic Factor[Title/Abstract])) OR (Mitogenic Factor, Lymphocyte[Title/Abstract])) OR (T-Cell Growth Factor[Title/Abstract])) OR (T Cell Growth Factor[Title/Abstract])) OR (T-Cell Stimulating Factor[Title/Abstract])) OR (T Cell Stimulating Factor[Title/Abstract])) OR (Thymocyte Stimulating Factor[Title/Abstract])) OR (Interleukin II[Title/Abstract])) OR (Ro 23 6019[Title/Abstract])) OR (RU 49637[Title/Abstract])) OR (RU-49637[Title/Abstract])) OR (RU49637[Title/Abstract]))) OR (("Interferon-gamma"[Mesh]) OR (((((((gamma-Interferon[Title/Abstract]) OR (Interferon, Immune[Title/Abstract])) OR (Immune Interferon[Title/Abstract])) OR (Type II Interferon[Title/Abstract])) OR (Interferon, Type II[Title/Abstract])) OR (Interferon Type II[Title/Abstract])) OR (Interferon, gamma[Title/Abstract])))) OR (("Tumor Necrosis Factor-alpha"[Mesh]) OR (((((((((Tumor Necrosis Factor alpha[Title/Abstract]) OR (Cachectin[Title/Abstract])) OR (Cachectin-Tumor Necrosis Factor[Title/Abstract])) OR (Cachectin Tumor Necrosis Factor[Title/Abstract])) OR (Tumor Necrosis Factor Ligand Superfamily Member 2[Title/Abstract])) OR (Tumor Necrosis Factor[Title/Abstract])) OR (TNF Superfamily, Member 2[Title/Abstract])) OR (TNFalpha[Title/Abstract])) OR (TNF-alpha[Title/Abstract])))) OR (("Interleukin-6"[Mesh]) OR ((((((((((((((((((((((((((((Interleukin 6[Title/Abstract]) OR (IL6[Title/Abstract])) OR (B-Cell Stimulatory Factor 2[Title/Abstract])) OR (B-Cell Stimulatory Factor-2[Title/Abstract])) OR (Differentiation Factor-2, B-Cell[Title/Abstract])) OR (Differentiation Factor 2, B Cell[Title/Abstract])) OR (B-Cell Differentiation Factor-2[Title/Abstract])) OR (B Cell Differentiation Factor 2[Title/Abstract])) OR (BSF-2[Title/Abstract])) OR (Hybridoma Growth Factor[Title/Abstract])) OR (Growth Factor, Hybridoma[Title/Abstract])) OR (IFN-beta 2[Title/Abstract])) OR (Plasmacytoma Growth Factor[Title/Abstract])) OR (Growth Factor, Plasmacytoma[Title/Abstract])) OR (Hepatocyte-Stimulating Factor[Title/Abstract])) OR (Hepatocyte Stimulating Factor[Title/Abstract])) OR (MGI-2[Title/Abstract])) OR (Myeloid Differentiation-Inducing Protein[Title/Abstract])) OR (Differentiation-Inducing Protein, Myeloid[Title/Abstract])) OR (Myeloid Differentiation Inducing Protein[Title/Abstract])) OR (B-Cell Differentiation Factor[Title/Abstract])) OR (B Cell Differentiation Factor[Title/Abstract])) OR (Differentiation Factor, B-Cell[Title/Abstract])) OR (Differentiation Factor, B Cell[Title/Abstract])) OR (IL-6[Title/Abstract])) OR (Interferon beta-2[Title/Abstract])) OR (Interferon beta 2[Title/Abstract])) OR (beta-2, Interferon[Title/Abstract])) OR (B Cell Stimulatory Factor-2[Title/Abstract])) OR (B Cell Stimulatory Factor 2[Title/Abstract])))) OR ((((((((((((((((((Interleukin 4[Title/Abstract]) OR (B-Cell Growth Factor-1[Title/Abstract])) OR (B Cell Growth Factor 1[Title/Abstract])) OR (B-Cell Growth Factor-I[Title/Abstract])) OR (B Cell Growth Factor I[Title/Abstract])) OR (B-Cell Proliferating Factor[Title/Abstract])) OR (B Cell Proliferating Factor[Title/Abstract])) OR (B-Cell Stimulating Factor-1[Title/Abstract])) OR (B Cell Stimulating Factor 1[Title/Abstract])) OR (B-Cell Stimulatory Factor 1[Title/Abstract])) OR (B-Cell Stimulatory Factor-1[Title/Abstract])) OR (BCGF-1[Title/Abstract])) OR (Binetrakin[Title/Abstract])) OR (BSF-

1[Title/Abstract])) OR (IL-4[Title/Abstract])) OR (IL4[Title/Abstract])) OR (Mast Cell Growth Factor-2[Title/Abstract])) OR (Mast Cell Growth Factor 2[Title/Abstract])) OR (B Cell Stimulatory Factor-1[Title/Abstract])) OR (B Cell Stimulatory Factor 1[Title/Abstract]))) OR (("Interleukin-5"[Mesh]) OR (((((((((((Interleukin 5[Title/Abstract]) OR (BCGF-II[Title/Abstract])) OR (Differentiation Factor, Eosinophil[Title/Abstract])) OR (T-Cell-Replacing Factor[Title/Abstract])) OR (T Cell Replacing Factor[Title/Abstract])) OR (IL-5[Title/Abstract])) OR (IL5[Title/Abstract])) OR (T-Cell Replacing Factor[Title/Abstract])) OR (Replacing Factor, T-Cell[Title/Abstract])) OR (B-Cell Growth Factor-II[Title/Abstract])) OR (B Cell Growth Factor II[Title/Abstract])) OR (Eosinophil Differentiation Factor[Title/Abstract])))) OR (("Interleukin-10"[Mesh]) OR ((((Interleukin 10[Title/Abstract]) OR (IL10[Title/Abstract])) OR (IL-10[Title/Abstract])) OR (Cytokine Synthesis Inhibitory Factor[Title/Abstract])))) Filters: from 2000/1/1 - 3000/12/12

Embase
Session Results
....

No.	Query Results	Results	Date
#34.	#32 AND #33	559	15 Mar 2021
#33.	[2000-2021]/py	24,234,152	15 Mar 2021
#32.	#30 AND #31	685	15 Mar 2021
#31.	#3 OR #8	8,223	15 Mar 2021
#30.	#11 OR #14 OR #17 OR #20 OR #23 OR #26 OR #29	778,748	15 Mar 2021
#29.	#27 OR #28	135,399	15 Mar 2021
#28.	'interleukin-10':ab,ti OR 'il10':ab,ti OR 'il-10':ab,ti OR 'cytokine synthesis inhibitory factor':ab,ti	96,319	15 Mar 2021
#27.	'interleukin 10'/exp	123,497	15 Mar 2021
#26.	#24 OR #25	34,991	15 Mar 2021
#25.	'interleukin-5':ab,ti OR 'bcgf-ii':ab,ti OR 'differentiation factor, eosinophil':ab,ti OR 't-cell-replacing factor':ab,ti OR 't cell replacing factor':ab,ti OR 'il-5':ab,ti OR 'il5':ab,ti OR 't-cell replacing factor':ab,ti OR 'replacing factor, t-cell':ab,ti OR 'b-cell growth factor-ii':ab,ti OR 'b cell growth factor ii':ab,ti OR 'eosinophil differentiation factor':ab,ti	22,381	15 Mar 2021
#24.	'interleukin 5'/exp	30,697	15 Mar 2021
#23.	#21 OR #22	97,650	15 Mar 2021
#22.	'interleukin-4':ab,ti OR 'b-cell growth factor-1':ab,ti OR 'b cell growth factor 1':ab,ti OR 'b-cell growth factor-i':ab,ti OR 'b cell growth factor i':ab,ti OR 'b-cell proliferating	69,176	15 Mar 2021

	factor':ab,ti OR 'b cell proliferating factor':ab,ti OR 'b-cell stimulating factor-1':ab,ti OR 'b cell stimulating factor 1':ab,ti OR 'b-cell stimulatory factor 1':ab,ti OR 'b-cell stimulatory factor-1':ab,ti OR 'bcgf-1':ab,ti OR 'binetrakin':ab,ti OR 'bsf-1':ab,ti OR 'il-4':ab,ti OR 'il4':ab,ti OR 'mast cell growth factor-2':ab,ti OR 'mast cell growth factor 2':ab,ti OR 'b cell stimulatory factor-1':ab,ti OR 'b cell stimulatory factor 1':ab,ti		
#21.	'interleukin 4'/exp	85,117	15 Mar 2021
#20.	#18 OR #19	288,356	15 Mar 2021
#19.	'interleukin-6':ab,ti OR 'il6':ab,ti OR 'b-cell stimulatory factor 2':ab,ti OR 'b-cell stimulatory factor-2':ab,ti OR 'differentiation factor-2, b-cell':ab,ti OR 'differentiation factor 2, b cell':ab,ti OR 'b-cell differentiation factor-2':ab,ti OR 'b cell differentiation factor 2':ab,ti OR 'bsf-2':ab,ti OR 'hybridoma growth factor':ab,ti OR 'growth factor, hybridoma':ab,ti OR 'ifn-beta 2':ab,ti OR 'plasmacytoma growth factor':ab,ti OR 'growth factor, plasmacytoma':ab,ti OR 'hepatocyte-stimulating factor':ab,ti OR 'hepatocyte stimulating factor':ab,ti OR 'mgi-2':ab,ti OR 'myeloid differentiation-inducing protein':ab,ti OR 'differentiation-inducing protein, myeloid':ab,ti OR 'myeloid differentiation inducing protein':ab,ti OR 'b-cell differentiation factor':ab,ti OR 'b cell differentiation factor':ab,ti OR 'differentiation factor, b-cell':ab,ti OR 'differentiation factor, b cell':ab,ti OR 'il-6':ab,ti OR 'interferon beta-2':ab,ti OR 'interferon beta 2':ab,ti OR 'beta-2, interferon':ab,ti OR 'b cell stimulatory factor-2':ab,ti OR 'b cell stimulatory factor 2':ab,ti	212,232	15 Mar 2021
#18.	'interleukin 6'/exp	262,617	15 Mar 2021
#17.	#15 OR #16	374,346	15 Mar 2021
#16.	'tumor necrosis factor alpha':ab,ti OR 'cachectin':ab,ti OR 'cachectin-tumor necrosis factor':ab,ti OR 'cachectin tumor necrosis	163,623	15 Mar 2021

	factor':ab,ti OR 'tumor necrosis factor ligand superfamily member 2':ab,ti OR 'tumor necrosis factor':ab,ti OR 'tnf superfamily, member 2':ab,ti OR 'tnfalpha':ab,ti OR 'tnf-alpha':ab,ti		
#15.	'tumor necrosis factor'/exp	334,856	15 Mar 2021
#14.	#12 OR #13	192,631	15 Mar 2021
#13.	'gamma-interferon':ab,ti OR 'interferon, immune':ab,ti OR 'immune interferon':ab,ti OR 'type ii interferon':ab,ti OR 'interferon, type ii':ab,ti OR 'interferon type ii':ab,ti OR 'interferon, gamma':ab,ti	30,725	15 Mar 2021
#12.	'gamma interferon'/exp	186,143	15 Mar 2021
#11.	#9 OR #10	128,186	15 Mar 2021
#10.	'interleukin-2':ab,ti OR 'il-2':ab,ti OR 'il2':ab,ti OR 'tcgf':ab,ti OR 'interleukine 2':ab,ti OR 'lymphocyte mitogenic factor':ab,ti OR 'mitogenic factor, lymphocyte':ab,ti OR 't-cell growth factor':ab,ti OR 't cell growth factor':ab,ti OR 't-cell stimulating factor':ab,ti OR 't cell stimulating factor':ab,ti OR 'thymocyte stimulating factor':ab,ti OR 'interleukin ii':ab,ti OR 'ro 23 6019':ab,ti OR 'ru 49637':ab,ti OR 'ru-49637':ab,ti OR 'ru49637':ab,ti	92,825	15 Mar 2021
#9.	'interleukin 2'/exp	100,181	15 Mar 2021
#8.	#6 AND #7	3,077	15 Mar 2021
#7.	tuberculous	56,461	15 Mar 2021
#6.	#4 OR #5	67,049	15 Mar 2021
#5.	'effusion, pleural':ab,ti OR 'effusions, pleural':ab,ti OR 'pleural effusions':ab,ti	12,780	15 Mar 2021
#4.	'pleura effusion'/exp	65,310	15 Mar 2021
#3.	#1 OR #2	5,579	15 Mar 2021
#2.	'effusion, malignant pleural':ab,ti OR 'effusions, malignant pleural':ab,ti OR 'malignant pleural effusion':ab,ti OR 'malignant pleural effusions':ab,ti OR 'pleural effusions, malignant':ab,ti	4,672	15 Mar 2021
#1.	'malignant pleura effusion'/exp	3,146	15 Mar 2021

....

2. NOS quality assessment:

Table S1a. The Newcastle-Ottawa Scale (NOS) quality assessment of the TNF-α included studies in this meta-analysis.

	**Selection**				**Comparability**	**Exposure**			**Overall NOS**
Study/Year	Is the case definition adequate	Representativeness of the cases	Selection of controls	Definition of controls	Comparability of cases and controls on the basis of the design or analysis	Ascertainment of exposure	Same method of ascertainment for cases and controls	Non-Response Rate	
Alemán et al. 2003	☆	☆		☆	☆	☆	☆		6
Ali et al. 2015	☆	☆		☆	☆☆	☆	☆		7
Ambade et al. 2011	☆			☆	☆	☆	☆		5
Atef et al. 2016	☆	☆		☆	☆☆	☆	☆		7
Budak et al. 2008	☆	☆		☆	☆	☆	☆		6
Ciledag et al. 2010	☆			☆	☆	☆	☆		5
Daniil et al. 2007	☆	☆		☆	☆	☆	☆		6
Gao et al. 2019	☆	☆		☆	☆☆	☆	☆		7
Hamed et al. 2004	☆	☆		☆	☆	☆	☆		6
Huang et al. 2008	☆	☆		☆	☆☆	☆	☆		7
Iglesias et al. 2005	☆	☆		☆	☆☆	☆	☆		7
Kiropoulos et al. 2007	☆	☆		☆	☆☆	☆	☆		7
Lee et al. 2010	☆	☆		☆	☆	☆	☆	☆	7
Li et al. 2014	☆	☆		☆	☆☆	☆	☆		7
Liu et al. 2020	☆	☆	☆	☆	☆☆	☆	☆		8

Momi et al. 2002	☆	☆		☆	☆☆	☆	☆		7
Qian et al. 2012	☆	☆		☆	☆	☆	☆		6
Wang et al. 2014	☆	☆		☆	☆	☆	☆		6
Yamada et al. 2001		☆		☆	☆	☆	☆	☆	6

Table S1b. The Newcastle-Ottawa Scale (NOS) quality assessment of the IFN-γ included studies in this meta-analysis.

	**Selection**				**Comparability**	**Exposure**			**Overall NOS**
Study/Year	Is the case definition adequate	Representativeness of the cases	Selection of controls	Definition of controls	Comparability of cases and controls on the basis of the design or analysis	Ascertainment of exposure	Same method of ascertainment for cases and controls	Non-Response Rate	
Ali et al. 2015	☆	☆		☆	☆	☆	☆		6
Ambade et al. 2011	☆			☆	☆	☆	☆		5
Budak et al. 2008		☆		☆	☆	☆	☆		5
Chen et al. 2001	☆	☆		☆	☆	☆	☆		6
Cui et al. 2010	☆	☆	☆	☆	☆☆	☆	☆		8
Ghayumi et al. 2011	☆	☆		☆	☆☆	☆	☆		7
Ibrahim et al. 2013	☆		☆	☆	☆☆	☆	☆		7

Krenke et al. 2008	☆	☆		☆	☆☆	☆	☆		7
Lee et al. 2013	☆	☆		☆	☆☆	☆	☆		7
Li et al. 2014	☆	☆		☆	☆	☆	☆		6
Marie et al. 2013	☆	☆		☆	☆☆	☆	☆		7
Okamoto et al. 2005 (1)	☆	☆		☆	☆	☆	☆		6
Okamoto et al. 2005 (2)	☆	☆		☆	☆	☆	☆		6
Valdés et al. 2009		☆		☆	☆	☆	☆		5
Valdés et al. 2014	☆	☆		☆	☆☆	☆	☆	☆	8
Wang et al. 2014	☆	☆		☆	☆	☆	☆	☆	7
Wu et al. 2013	☆	☆		☆	☆☆	☆	☆		7
Xue et al. 2007	☆	☆		☆	☆	☆	☆		6
Yurt et al. 2014	☆	☆		☆	☆☆	☆	☆		7
Zhang et al. 2017		☆		☆	☆	☆	☆		5

Table S1c. The Newcastle-Ottawa Scale (NOS) quality assessment of the IL-2 included studies in this meta-analysis.

	**Selection**				**Comparability**	**Exposure**			**Overall NOS**
Study/Year	Is the case definition adequate	Representativeness of the cases	Selection of controls	Definition of controls	Comparability of cases and controls on the basis of the design or analysis	Ascertainment of exposure	Same method of ascertainment for cases and	Non-Response Rate	

							Same method of ascertainment for cases and controls		
Cui et al. 2010	☆	☆	☆	☆	☆☆	☆	☆		8
Zhang et al. 2020	☆	☆		☆	☆	☆	☆		6
Shu et al. 2015	☆	☆		☆	☆	☆	☆		6

Table S1d. The Newcastle-Ottawa Scale (NOS) quality assessment of the IL-4 included studies in this meta-analysis.

	**Selection**				**Comparability**	**Exposure**			**Overall NOS**
Study/Year	Is the case definition adequate	Representativeness of the cases	Selection of controls	Definition of controls	Comparability of cases and controls on the basis of the design or analysis	Ascertainment of exposure	Same method of ascertainment for cases and controls	Non-Response Rate	
Budak et al. 2008		☆		☆	☆	☆	☆		5
Chen et al. 2016	☆	☆		☆	☆	☆	☆	☆	7
Cui et al. 2010	☆	☆	☆	☆	☆☆	☆	☆		8
Ghayumi et al. 2011	☆	☆		☆	☆☆	☆	☆		7
Okamoto et al. 2005	☆	☆		☆	☆	☆	☆		6
Zhang et al. 2020	☆	☆		☆	☆	☆	☆		6

Table S1e. The Newcastle-Ottawa Scale (NOS) quality assessment of the IL-10 included studies in this meta-analysis.

Study/Year	Selection				Comparability	Exposure			Overall NOS
	Is the case definition adequate	Representativeness of the cases	Selection of controls	Definition of controls	Comparability of cases and controls on the basis of the design or analysis	Ascertainment of exposure	Same method of ascertainment for cases and controls	Non-Response Rate	
Budak et al. 2008		☆		☆	☆	☆	☆		5
Chen et al. 2001	☆	☆		☆	☆	☆	☆		6
Chen et al. 2016	☆	☆		☆	☆	☆	☆	☆	7
Ciledag et al. 2010	☆			☆	☆☆	☆	☆		6
Ghayumi et al. 2011	☆	☆		☆	☆☆	☆	☆		7
Li et al. 2014	☆	☆		☆	☆	☆	☆		6
Shu et al. 2015	☆	☆		☆	☆	☆	☆		6
Zhang et al. 2020	☆	☆		☆	☆	☆	☆		6

Table S1f. The Newcastle-Ottawa Scale (NOS) quality assessment of the IL-6 included studies in this meta-analysis.

Study/Year	**Selection**				**Comparability**	**Exposure**			**Overall NOS**
	Is the case definition adequate	Representativeness of the cases	Selection of controls	Definition of controls	Comparability of cases and controls on the basis of	Ascertainment of exposure	Same method of ascertainment for	Non-Response Rate	

				the design or analysis		cases and controls		
Chen et al. 2016	☆	☆	☆	☆	☆	☆	☆	7
Dalil et al. 2021	☆	☆	☆	☆	☆	☆		6
Daniil et al. 2007	☆	☆	☆	☆	☆	☆		6
Ferreiro et al. 2019	☆	☆	☆	☆☆	☆	☆		7
Kiropoulos et al. 2007	☆	☆	☆	☆☆	☆	☆		7
Marie et al. 2013	☆	☆	☆	☆☆	☆	☆		7
Qian et al. 2012	☆	☆	☆	☆	☆	☆		6
Sayed et al. 2015	☆	☆	☆	☆	☆	☆		6
Shu et al. 2015	☆	☆	☆	☆	☆	☆		6
Teixeira et al. 2016	☆	☆	☆	☆	☆	☆		6
Xirouchaki et al. 2002	☆	☆	☆	☆☆	☆	☆		7
Zhang et al. 2020	☆	☆	☆	☆	☆	☆		6

3. Characteristics of studies included:

Table S2a. Characteristics of included TNF-α studies.

**Study**	**Country**	**Age**	**Type of study**	**Assay**	**Design**	**Etiology**	**Histopathology**	**NOS**
Alemán et al. 2003	Spain	NS	Prospective	ELISA	Cohort study	MPE: diverse	NS	6
Ali et al. 2015	Egypt	MPE: 54.50 ± 10.59	Prospective	ELISA	Cohort study	MPE: lung cancer	MPE: diverse	7

		TPE: 36.95 ± 12.77						
Ambade et al. 2011	India	NS	Prospective	ELISA	Cohort study	MPE: diverse	NS	5
Atef et al. 2016	Egypt	MPE: 67.6 ± 10.4 TPE: 50.3 ± 15.1	Prospective	ELISA	Cohort study	NS	NS	7
Budak et al. 2008	Turkey	MPE: 61.4 ± 13.5 TPE:42.7 ± 19.7	Prospective	ELISA	Cohort study	MPE: diverse	NS	6
Ciledag et al. 2010	Turkey	NS	Prospective	ELISA	Cohort study	NS	NS	5
Daniil et al. 2007	Greece	Mean: 65	Prospective	ELISA	Cohort study	MPE: diverse	NS	6
Gao et al. 2019	China	18-72	Prospective	ELISA	Cohort study	NS	NS	7
Hamed et al. 2004	Egypt	MPE: 34.9 ± 8.7 TPE: 29.5 ± 7.4	Prospective	ELISA	Cohort study	MPE: diverse	NS	6
Huang et al. 2008	China	MPE: 34-78 TPE: 17-81	Prospective	ELISA	Cohort study	MPE: lung cancer	MPE: diverse	7
Iglesias et al. 2005	Spain	18–94	Retrospective	ELISA	Case-control study	NS	NS	7
Kiropoulos et al. 2007	Greece	MPE: 70±11 TPE: 55±16.5	Prospective	ELISA	Cohort study	NS	NS	7
Lee et al. 2010	Korea	28-75	Retrospective	ELISA	Case-control study	MPE: lung cancer	NS	7
Li et al. 2014	China	MPE: 55.5 ± 12.4 TPE: 51.3 ± 11.8	Prospective	ELISA	Cohort study	MPE: diverse	NS	7
Liu et al. 2020	China	MPE: 60.48 ± 9.20 TPE: 57.72	Prospective	ELISA	Cohort study	NS	NS	8

		± 15.68						
Momi et al. 2002	Japan	Mean: 63.1 ± 35.2	Prospective	ELISA	Cohort study	NS	NS	7
Qian et al. 2012	China	<65: 34 ≥65: 45	Prospective	ELISA	Cohort study	MPE: lung cancer	MPE: adenocarcinoma	6
Wang et al. 2014	China	MPE: 55.52±16.51 TPE: 35.46±18.32	Retrospective	ELISA	Case-control study	NS	NS	6
Yamada et al. 2001	Japan	MPE: 74 TPE: 68	Prospective study	ELISA	Cohort study	MPE: diverse	NS	6

ELISA, enzyme-linked immunosorbent assay; NS, Not specified.

Table S2b. Characteristics of included IFN-γ studies.

**Study**	**Country**	**Age**	**Type of study**	**Assay**	**Design**	**Etiology**	**Histopathology**	**NOS**
Ali et al. 2015	Egypt	MPE: 54.50 ± 10.59 TPE: 36.95 ± 12.77	Prospective	ELISA	Cohort study	MPE: lung cancer	MPE: diverse	6
Ambade et al. 2011	India	NS	Prospective	ELISA	Cohort study	MPE: diverse	NS	5
Budak et al. 2008	Turkey	MPE: 61.4 ± 13.5 TPE:42.7 ± 19.7	Prospective	ELISA	Cohort study	MPE: diverse	NS	5
Chen et al. 2001	China	MPE: 67.9 ± 11.2 TPE: 67.6 ± 12.2	Retrospective	ELISA	Case-control study	MPE: lung cancer	MPE: diverse	6
Cui et al. 2010	China	MPE: 54 ± 11 TPE: 38 ± 17	Retrospective	ELISA	Case-control study	MPE: lung cancer	NS	8
Ghayumi et al. 2011	Iran	MPE: 65.5 ± 15.1 TPE: 64.5	Prospective	ELISA	Cohort study	MPE: lung cancer	MPE: diverse	7

		± 15.2						
Ibrahim et al. 2013	Egypt	MPE: 43 (37–68) TPE: 35.5 (29–53)	Prospective	ELISA	Cohort study	MPE: lung cancer	MPE: adenocarcinoma	7
Krenke et al. 2008	Poland	MPE: 46.7 ± 20.1 TPE: 68.1 ± 9.8	Prospective	ELISA	Cohort study	NS	NS	7
Lee et al. 2013	Korea	MPE: 69 ± 14 TPE: 55 ± 23	Retrospective	ELISA	Case-control study	MPE: diverse	MPE: diverse	7
Li et al. 2014	China	MPE: 55.5 ± 12.4 TPE: 51.3 ± 11.8	Prospective	ELISA	Cohort study	MPE: diverse	NS	6
Marie et al. 2013	Kingdom of Saudi Arabia	NS	Prospective	ELISA	Cohort study	NS	NS	7
Okamoto et al. 2005 (1)	Japan	MPE: 74 (53–88) TPE: 70 (50–94)	Retrospective	ELISA	Case-control study	MPE: lung cancer	NS	6
Okamoto et al. 2005 (2)	Japan	MPE: 69 (50–94) TPE: 74 (53–88)	Prospective	ELISA	Cohort study	MPE: lung cancer	NS	6
Valdés et al. 2009	Spain	MPE: 63.7 ± 12.8 TPE: 39.7 ±20	Prospective	ELISA	Cohort study	MPE: diverse	NS	5
Valdés et al. 2014	Spain	MPE: 67.3 ± 13.4 2. 36.95 ± 12.78"	Prospective	ELISA	Cohort study	MPE: diverse	NS	8
Wang et al. 2014	China	MPE: 55.52 ± 16.51 TPE: 35.46 ± 18.32	Retrospective	ELISA	Case-control study	NS	NS	7
Wu et al. 2013	China	MPE: 57.78± 2.09	Prospective	ELISA	Cohort study	MPE: lung	MPE: diverse	7

		TPE: 40.10 ± 2.40				cancer		
Xue et al. 2007	China	MPE: 51.4 ± 9.8 TPE: 48.5 ± 10.6	Retrospective	ELISA	Case-control study	MPE: diverse	NS	6
Yurt et al. 2014	Turkey	MPE: 65 (50–68) TPE: 25 (20–37)	Prospective	ELISA	Cohort study	NS	NS	7
Zhang et al. 2017	China	MPE: 45.8 ± 2.7 TPE: 45.6 ± 2.5	Retrospective	ELISA	Case-control study	MPE: diverse	NS	5

ELISA, enzyme-linked immunosorbent assay; NS, Not specified.

Table S2c. Characteristics of included IL-2 studies.

**Study**	**Country**	**Age**	**Type of study**	**Assay**	**Design**	**Etiology**	**Histopathology**	**NOS**
Cui et al. 2010	China	MPE: 54 ± 11 TPE: 38 ± 17	Retrospective	ELISA	Case-control study	MPE: lung cancer	NS	8
Shu et al. 2015	China	MPE: 67.3±14.6 TPE: 66.5±18.9	Prospective	Luminex	Cohort study	NS	NS	6
Zhang et al. 2020	China	MPE: 51 (36–80) TPE: 45 (21–76)	Retrospective	CBA	Case-control study	MPE: diverse	NS	6

CBA, cytometric bead array; ELISA, enzyme-linked immunosorbent assay; NS, Not specified.

Table S2d. Characteristics of included IL-4 studies.

**Study**	**Country**	**Age**	**Type of study**	**Assay**	**Design**	**Etiology**	**Histopathology**	**NOS**
Budak et al. 2008	Turkey	MPE: 61.4 ± 13.5 TPE:42.7 ± 19.7	Prospective	ELISA	Cohort study	MPE: diverse	NS	5

Chen et al. 2016	China	MPE: 67.9±13.1 TPE: 63.0±21.9	Prospective	CBA	Cohort study	NS	NS	7
Cui et al. 2010	China	MPE: 54 ± 11 TPE: 38 ± 17	Retrospective	ELISA	Case-control study	MPE: lung cancer	NS	8
Ghayumi et al. 2011	Iran	MPE: 65.5 ± 15.1 TPE: 64.5 ± 15.2	Prospective	ELISA	Cohort study	MPE: lung cancer	MPE: diverse	7
Okamoto et al. 2005	Japan	MPE: 74 (53–88) TPE: 70 (50–94)	Retrospective	ELISA	Case-control study	MPE: lung cancer	NS	6
Zhang et al. 2020	China	MPE: 51 (36–80) TPE: 45 (21–76)	Retrospective	CBA	Case-control study	MPE: diverse	NS	6

CBA, cytometric bead array; ELISA, enzyme-linked immunosorbent assay; NS, Not specified.

Table S2e. Characteristics of included IL-10 studies.

**Study**	**Country**	**Age**	**Type of study**	**Assay**	**Design**	**Etiology**	**Histopathology**	**NOS**
Budak et al. 2008	Turkey	MPE: 61.4 ± 13.5 TPE:42.7 ± 19.7	Prospective	ELISA	Cohort study	MPE: diverse	NS	5
Chen et al. 2001	China	MPE: 67.9 ± 11.2 TPE: 67.6 ± 12.2	Retrospective	ELISA	Case-control study	MPE: lung cancer	MPE: diverse	6
Chen et al. 2016	China	MPE: 67.9±13.1 TPE: 63.0±21.9	Prospective	CBA	Cohort study	NS	NS	7
Ciledag et al. 2010	Turkey	NS	Prospective	ELISA	Cohort study	NS	NS	6
Ghayumi et al. 2011	Iran	MPE: 65.5 ± 15.1 TPE: 64.5	Prospective	ELISA	Cohort study	MPE: lung cancer	MPE: diverse	7

		± 15.2						
Li et al. 2014	China	MPE: 55.5 ± 12.4 TPE: 51.3 ± 11.8	Prospective	ELISA	Cohort study	MPE: diverse	NS	6
Shu et al. 2015	China	MPE: 67.3±14.6 TPE: 66.5±18.9	Prospective	Luminex	Cohort study	NS	NS	6
Zhang et al. 2020	China	MPE: 51 (36–80) TPE: 45 (21–76)	Retrospective	CBA	Case-control study	MPE: diverse	NS	6

CBA, cytometric bead array; ELISA, enzyme-linked immunosorbent assay; NS, Not specified.

Table S2f. Characteristics of included IL-6 studies.

**Study**	**Country**	**Age**	**Type of study**	**Assay**	**Design**	**Etiology**	**Histopathology**	**NOS**
Chen et al. 2016	China	MPE: 67.9±13.1 TPE: 63.0±21.9	Prospective	CBA	Cohort study	NS	NS	7
Dalil et al. 2021	Iran	MPE: 59.5 ± 2.8 TPE: 52.3 ± 3.9	Prospective	ELISA	Cohort study	NS	MPE: diverse	6
Daniil et al. 2007	Greece	Mean: 65	Prospective	ELISA	Cohort study	MPE: diverse	NS	6
Ferreiro et al. 2019	Spain	MPE: 72 (62-80) TPE: 34 (28.3-58.5)	Prospective	CLEIA	Cohort study	MPE: diverse	NS	7
Kiropoulos et al. 2007	Greece	MPE: 70 ± 11 TPE: 55 ± 16.5	Prospective	ELISA	Cohort study	NS	NS	7
Marie et al. 2013	Kingdom of Saudi Arabia	NS	Prospective	ELISA	Cohort study	NS	NS	7
Qian et al. 2012	China	<65: 34 ≥65: 45	Prospective	ELISA	Cohort study	MPE: lung	MPE: adenoca	6

						cancer	rcinoma	
Sayed et al. 2015	Egypt	MPE: 65 ± 9.8 TPE: 53.1 ± 6.94	Prospective	ELISA	Cohort study	MPE: diverse	MPE: adenocarcinoma	6
Shu et al. 2015	China	MPE: 67.3±14.6 TPE: 66.5±18.9	Prospective	Luminex	Cohort study	NS	NS	6
Teixeira et al. 2016	Brazil	MPE: 62 (21–83) TPE: 35 (16–81)	Prospective	ELISA	Cohort study	MPE: diverse	NS	6
Xirouchaki et al. 2002	Greece	61 ± 14	Prospective	RIA	Cohort study	NS	NS	7
Zhang et al. 2020	China	MPE: 51 (36–80) TPE: 45 (21–76)	Retrospective	CBA	Case-control study	MPE: diverse	NS	6

CBA, cytometric bead array; CLEIA, chemiluminescent enzyme immunoassay; ELISA, enzyme-linked immunosorbent assay; NS, not specified; RIA, radioimmunoassay.

4. Publication bias:

Figure S3a. A. Funnel plots for TNF-α.

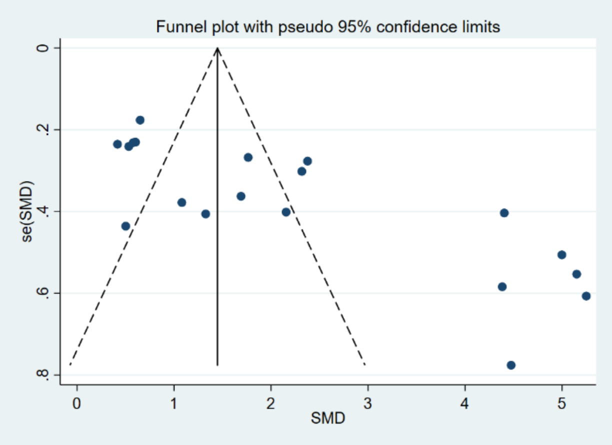

Funnel plot and Begg's test of effect sizes for publication bias.

Begg's Test

adj. Kendall's Score (P-Q) = 85
Std. Dev. of Score = 28.58
Number of Studies = 19
z = 2.97
Pr > |z| = 0.003

z = 2.94 (continuity corrected)

Pr > |z| = 0.003 (continuity corrected)

SMD, standardized mean difference; SE, standard error; Std_ Dev, standard deviation.

Figure S3b. Funnel plots with trim and fill method.

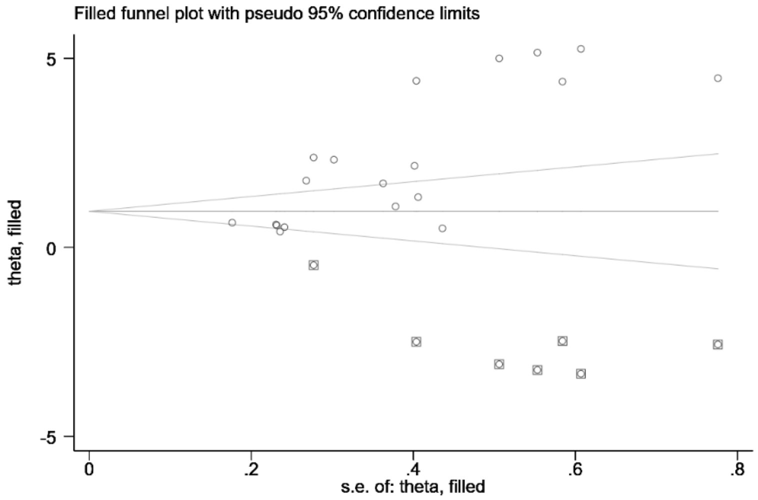

Funnel plot for TNF-α, adjusted with trim and fill method Circles stand for included studies; diamonds stand for presumed missing studies.

Figure S3c. A. Funnel plots for IFN-γ.

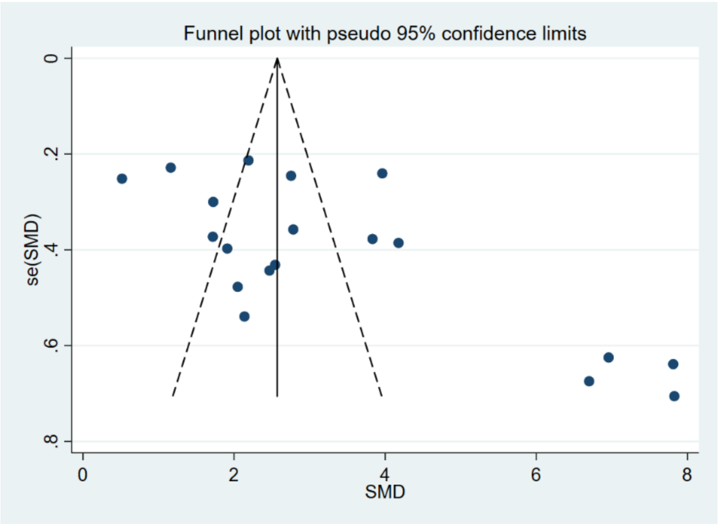

Begg's Test

adj. Kendall's Score (P-Q) = 71

Std. Dev. of Score = 28.58

Number of Studies = 19

z = 2.48

Pr > |z| = 0.013

z = 2.45 (continuity corrected)

Pr > |z| = 0.014 (continuity corrected)

Figure S3d. Funnel plots with trim and fill method.

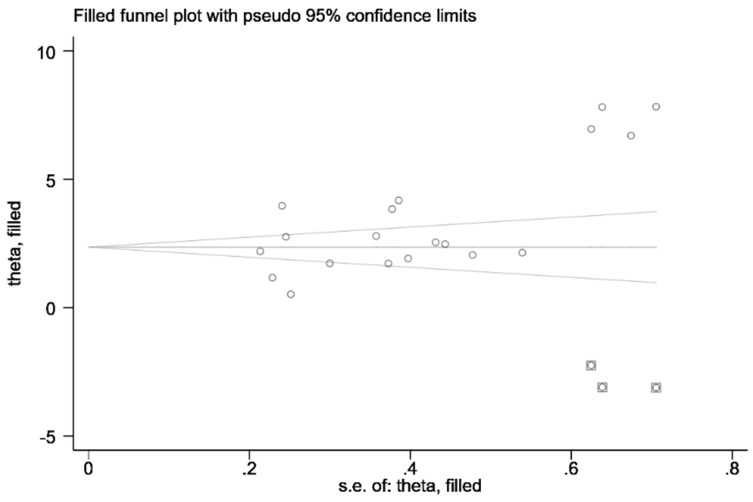

Figure S3e. Funnel plots for IL-6.

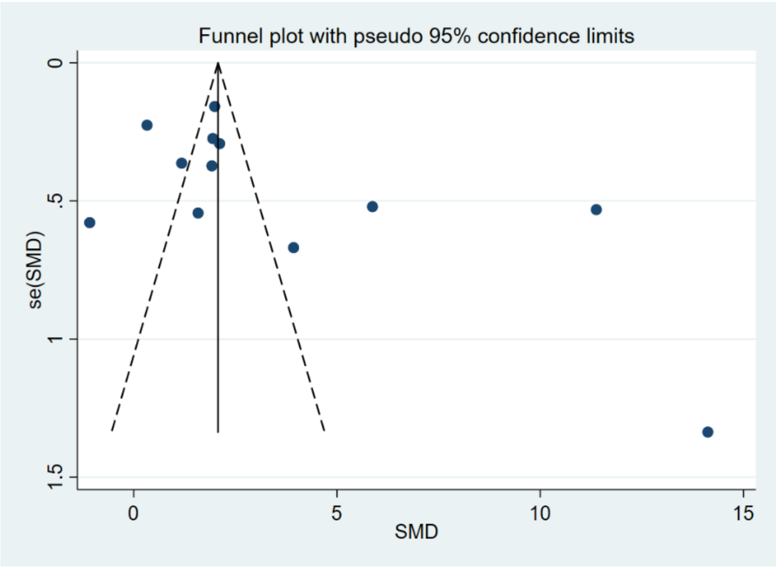

Begg's Test

adj. Kendall's Score (P-Q) = 22

Std. Dev. of Score = 14.58

Number of Studies = 12

z = 1.51

Pr > |z| = 0.131

z = 1.44 (continuity corrected)

Pr > |z| = 0.150 (continuity corrected)
