# Supplementary material for: A meta-analysis of Th1 and Th2 cytokine profiles differentiating tuberculous from malignant pleural effusion
Source: Sci Rep. 2022 Feb 17;12:2743. doi: 10.1038/s41598-022-06685-8 (PMC8854582; doi:10.1038/s41598-022-06685-8)
Supplement: Supplementary file 3 — Supplementary Tables. [file 41598_2022_6685_MOESM3_ESM.pdf]

Table S1. Stratified analysis of pooled SMD for TNF-α.

Stratified analysis	Pooled SMD (95% CI)	Heterogeneity	Meta regression (p-value)
**Country**			0.325
Asian	1.49 (0.85-2.13)	94%, $p < 0.001$	
Non-Asian	2.14 (1.47-2.82)	71%, $p < 0.01$	
**Study design**			0.500
Cohort study	1.58 (1.09, 2.08)	89%, $p < 0.001$	
Case-control study	2.43 (-1.33, 6.19)	98%, $p < 0.001$	
**Etiology**			0.779
Lung cancer	1.43 (0.46, 2.41)	88%, $p < 0.001$	
Diverse	1.70 (0.73, 2.67)	92%, $p < 0.001$	
**Publishing year**			0.462
≤2010	1.43 (0.76, 2.09)	90%, $p < 0.001$	
>2010	1.99 (1.11, 2.87)	94%, $p < 0.001$	
**Quality score**			0.699
≤6	1.88 (0.93, 2.82)	93%, $p < 0.001$	
>6	1.52 (0.87, 2.17)	91%, $p < 0.001$	

CI, Confidence Intervals; NA: not available.

Table S2. Stratified analysis of pooled SMD for IFN-γ.

Stratified analysis	Pooled SMD (95% CI)	Heterogeneity	Meta regression (p-value)
**Country**			0.669
Asian	3.17 (2.30, 4.04)	96%, $p < 0.001$	
Non-Asian	3.67 (2.49, 4.85)	92%, $p < 0.001$	
**Study design**			0.834
Cohort study	3.38 (2.47, 4.29)	95%, $p < 0.001$	
Case-control study	3.16 (1.77, 4.54)	97%, $p < 0.001$	
**Etiology**			0.829
Lung cancer	3.11 (1.84, 4.39)	96%, $p < 0.001$	
Diverse	3.39 (2.24, 4.53)	96%, $p < 0.001$	
**Publishing year**			0.743
≤2010	3.12 (1.98, 4.26)	95%, $p < 0.001$	
>2010	3.45 (2.43, 4.47)	96%, $p < 0.001$	
**Quality score**			0.502
≤6	2.94 (2.01, 3.88)	93%, $p < 0.001$	
>6	3.65 (2.49, 4.82)	97%, $p < 0.001$	

CI, Confidence Intervals.

Table S3. Stratified analysis of pooled SMD for IL-6.

Stratified analysis	Pooled SMD (95% CI)	Heterogeneity	Meta regression (p-value)
**Assay**			0.941
ELISA	3.28 (1.69-4.87)	96%, p < 0.001	
Others	3.78 (1.07-6.49)	99%, p < 0.001	
**Country**			0.823
Asian	2.66 (1.43-3.89)	96%, p < 0.001	
Non-Asian	3.99 (0.97-7.01)	99%, p < 0.001	
**Study design**			0.708
Cohort study	3.73 (2.01-5.46)	98%, p < 0.001	
Case-control study	1.99 (1.68-2.30)	NA	
**Etiology**			0.713
Lung cancer	1.93 (1.39-2.47)	NA	
Diverse	4.00 (0.82-7.18)	99%, p < 0.001	
**Publishing year**			0.659
≤2010	2.42 (1.56-3.28)	70%, p =0.04	
>2010	3.90 (2.09-5.72)	98%, p < 0.001	
**Quality score**			0.845
≤6	3.04 (1.64-4.45)	97%, p < 0.001	
>6	3.98 (0.68-7.28)	99%, p < 0.001	

CI, Confidence Intervals; ELISA, enzyme-linked immunosorbent assay; NA: not available.
